# Headspace solid-phase microextraction method for extracting volatile constituents from the different parts of Saudi *Anethum graveolens* L. and their antimicrobial activity

**DOI:** 10.1016/j.heliyon.2022.e09051

**Published:** 2022-03-05

**Authors:** Hanan Y. Aati, Shagufta Perveen, Sultan Aati, Raha Orfali, Jawaher H. Alqahtani, Areej M. Al-Taweel, Juergen Wanner, Abdulrahman Y. Aati

**Affiliations:** a Department of Pharmacognosy, College of Pharmacy, King Saud University, P.O. Box 2457, Riyadh 11451, Saudi Arabia; b Department of Chemistry, School of Computer, Mathematical and Natural Sciences, Morgan State University, Baltimore, MD 21251, USA; c UWA Dental School, University of Western Australia, 17Monash Avenue, Nedland WA 6009, Australia; d Kurt Kitzing Co., Hinterm Alten Schloss 21, D-86757 Wallerstein, Germany; e Rokn Al-Madaein Pharmaceutical Warehouse Co., Riyadh, Saudi Arabia

**Keywords:** *A. graveolens*, Dill, Chemical composition, Antimicrobial, Antifungal

## Abstract

*Anethum graveolens* L. is a famous aromatic herb that is widely used as a spice and has been applied in folk medicine to cure many diseases. The current work was carried out to compare the chemical composition and antimicrobial potency of essential oils obtained from the different parts of Saudi *Arabia. graveolens*. The oil constituents were extracted by headspace solid-phase microextraction and were quantified and qualitatively identified using GC/MS. As a result, essential oil isolated from *A. graveolens* seeds exhibited the highest antimicrobial activity compared to oils isolated from other parts, followed by flowers, leaves and stems. All tested *A. graveolens* essential oil samples exhibited stronger antifungal activities against *Aspergillus parasiticus* when compared to itraconazole. To the best of our knowledge, the current work is the first report comparing different parts of Saudi *A. graveolens* plant with respect to their essential oil chemical composition and antimicrobial potentials. The essential oil of *A. graveolens* seeds have the highest contents of carvone and limonene and show superior antimicrobial activities compared to other parts of the plant.

## Highlights

• Headspace Solid-phase Microextraction (HS-SPME) is highly effective for analyzing volatile constituents.
• The essential oil of Dill plays a critical role in its antimicrobial activity.
• HS-SPME method can be successfully applied for extracting and separating volatile constituents in different parts of *A. graveolens* plants and more effective for identifying and collecting more constituents.

## Graphical abstract

**Figure:**
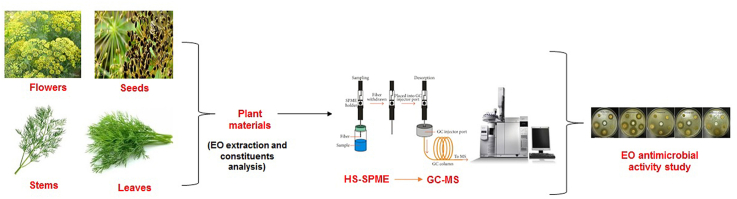

## 1 Introduction

*Anethum graveolens* L. (AG) is an important aromatic annual herb that is widely used as a spice in addition to its well-known carminative, stomachic and diuretic applications. It is a member of the Apiaceae family, which is distributed in Southwest Asia and the Mediterranean and is native to Southeast Europe. It is commonly known as dill in English and “Shabat شبت” in Arabic (Navneet et al., 2019). It has been cultivated since ancient times and found to be closely related to the species of Indian dill (*A. sowa*) and European dill (*A. graveolens*). Mediterranean countries in Eastern Europe, India and Russia are the main producers of dill essential oil (Henry et al., 2015).

The seeds of *A. graveolens* are used in folk medicine as an appetizer, carminative, diuretic, stomachic, digestive, sedative and in hemorrhoids (Jana and Shekhawat, 2010; Singh et al., 2005). Chewing of the seeds improves bad breath, e.g., halitosis. *A. graveolens* stimulates milk flow in lactating/nursing mothers and is often given to livestock for this reason. It also cures mental disorders, urinary infections and piles (Zheng et al., 1992). This plant is one of the additives used in gripe water usually given for relief of colic pain and hiccups in babies/infants and flatulence in young children (Nautiyal and Tiwari., 2011). Furthermore, it is used as a natural flavor enhancer in many food items, particularly in sauces, salads, soups, sea foods, fried meats and mainly in pickles. The essential oil is extracted from leaves, seeds and stems and is used as a flavoring agent in food and beverages due to its pleasant aroma. Additionally, the essential oil is used as perfume to aromatize soaps, detergents and cosmetics (Leopold et al., 2003).

Phytochemical screening of this plant showed that its flowers, seeds, leaves and stems were rich in polyphenols, tannins, terpenes, and cardiac and flavonoid glycosides (Singh et al., 2005; Nautiyal and Tiwari., 2011). Various pharmacological activities have been reported from *A. graveolens* plant parts, such as antihypercholesterolemic, chemopreventive effects, antimicrobial, antioxidant, antihyperlipidemic, antiulcer, mucosal protective, antisecretory, anticancer, antidiabetic, anti-inflammatory, insecticidal and analgesic activities (Zheng et al., 1992).

The *A. graveolens* volatile oil was reported to have numerous pharmacological activities, including antioxidant (El Mansouri et al., 2016), anticancer (Mohammed et al., 2018), antibacterial, antifungal and antimicrobial activities (Chahal et al., 2017), diuretic (Sahib et al., 2012), antidiabetic (Goodarzi et al., 2016), and anti-inflammatory & analgesic (Naseri et al., 2012) activities. The essential oil of *A. graveolens* seeds growing in Saudi Arabia were analyzed using HS-SPME and reported previously (Al-Massarani et al., 2018). To the best of our knowledge, no previous study has been conducted to compare the essential oil content of different parts of *A. graveolens* growing in Saudi Arabia using HS-SPME. The aim of our study was to evaluate and compare the volatile oil profiles of the flowers, seeds, leaves and stems of *A. graveolens* growing in Saudi Arabia and to determine the antimicrobial activities of each of these parts.

The headspace solid-phase microextraction (HS-SPME) technique is a relatively new method for the extraction of volatiles, was developed in 1993 and has attracted growing attention over the past decade. It utilizes a fine quartz fiber with a polymeric coating to extract organic compounds from its matrix and directly transfer them into the injector of a gas chromatograph for thermal desorption and analysis. HS-SPME is designed to extract volatile compounds with a wide range of boiling points without forming artifacts. In the current work, the essential oils of the seeds, flowers, leaves and stems of Saudi *A. graveolens* plants were separately extracted using HS-SPME and analyzed using GC/MS. Subsequently, the antimicrobial capacity of each essential oil sample was evaluated against various bacterial and fungal strains.

## 2 Materials and method

### 2.1 Plant material

The aerial part of wild *A. graveolens* at the flowering stage was collected from Jizan city of Saudi Arabia. In March 2019, the seeds, flowers, stems, and leaves were carefully separated and then dried in the shade. It was identified by plant taxonomist Dr. Rajakrishnan Rajagopal, a botanist of the Science College Herbarium, King Saud University. A voucher specimen (#24547) was deposited in the herbarium of the College of Science, KSU. All protocols involving plants adhered to relevant ethical guidelines/regulations of King Saud University (Article #14).

### 2.2 Headspace solid-phase microextraction

Plant materials were placed in a 5 ml headspace vial and enriched for 1 h on a SPME fiber (PDMS/DVB/Carboxen, SUPELCO part no. 57298-U) at a temperature of 80 °C in a metal block in such a way that the plant material was subjected to the elevated temperature while the SPME fiber was kept cold (room temperature). The enriched fiber was placed in the GC injector, and the fiber desorbed for 1 min at 250 °C.

### 2.3 GC–FID and GC–MS analysis

Gas chromatography–flame ionization detector (GC–FID) and GC–MS analyses were performed in one run using an MS-FID-splitter consisting of a quartz Y-splitter, a short (ca. 20 cm) 0.1 mm id fused silica restrictor column as an inlet to the GC–MS interface, and a ca. A 1 m × 0.25 mm deactivated fused silica column served as a transfer line to the FID detector. The restrictor column was used to limit the MS vacuum flow and prevent the insertion of combustion gases from the FID, which operated at atmospheric pressure. Moreover, the analytical column flow had to be greater than the inflow to the MS detector, which was limited to approximately 1 mL/min by the restriction line. The GC column flow should also be constant; otherwise, the FID/MS split ratio would change with temperature. This configuration yielded an FID and MS chromatogram with almost identical retention times (RTs), thus facilitating the assignment of FID peaks to each substance. The Thermo Fisher Scientific Trace GC Ultra used a split/split less injector heated at 250 °C and connected to a 50 m × 0.25 mm × 1.0 μm SE-52 (95% polydimethylsiloxane, 5% polydiphenylsiloxane) capillary column (prepared and tested for deactivation and separation efficiency in our lab, Kurt, 1986), an FID detector operating at 250 °C, and a TriPlus RSH Autosampler.

For essential oil component identification, a Thermo Fisher Scientific ISQ mass spectrometer connected with a GC/MS interface heated at 250 °C was used in electron ionization mode at 70 eV and a filament at 50 μA. Furthermore, an ion source operating at 230 °C and a scan range of 40–500 amu. The oven temperature use gradient program involved heating for 1 min at 60 °C, then heating increased to 230 °C at a rate of 3 °C/min, and a 230 °C isotherm for 12.3 min. The carrier gas was helium and flowed at a constant rate of 1.5 mL/min.

### 2.4 Identification of the essential oil components

Thermo Xcalibur 2.2 software was used to identify the EO compounds by correlating the obtained mass spectra with the databases of the National Institute of Standards and Technology (The NIST 08), Wiley 8^th^ ed (Wiley Registry™), Adams library (Robert, 2007), Mass Finder terpenoids library, and our own library. However, the mass spectra's simple comparison is not sufficient for a clear identification, especially for sesquiterpenes. Thus, the chromatogram position was also considered a second criterion determined by comparing the calculated retention indices (RIs) of the peaks with the corresponding literature data (Wiley Registry™ & The NIST 08) or reference compounds. The RI values were determined by measuring the RTs of a series of *n*-alkanes that were eluted across the entire chromatogram and were calculated according to the method of Van den Dool and Kratz (Van and Kratz 1963). Moreover, the EO components were quantified using normalized peak area calculations of the FID chromatogram without (by first approximation) relative FID-response factors.

### 2.5 Antibacterial assay

Agar diffusion methods were used in this study to assay the antibacterial activity of the essential oils against 9 different microbial strains, including Gram-positive; *Staphylococcus aureus* (CP011526.1), *Bacillus licheniformis* (KX785171.1) and *Listeria innocua* (DSM, 20649) and the Gram-negative; *Enterobacter xiangfangensis* (CP017183.1), *Escherichia fergusonii* (CU928158.2) and *Pseudomonas aeruginosa* (NR-117678.1) bacterial strains. TSB tryptone soya broth media were used previously to grow the microorganisms as mentioned above for 24 h. Nutrient agar plates were used to distribute 0.1 mL of the microbial suspensions. Ten and 20 μl of each essential oil were spotted on the inoculated plates. Under sterile conditions and after 15 min, plates were incubated under optimal growth conditions for each strain. The free area without microbial growth was measured three times to detect the diameter of the zone of inhibition, and the mean was calculated. Gentamycin (10 μg) and tetracycline (10 μg) were used as positive controls, while DMSO was used as a negative control.

### 2.6 Antifungal activity

All essential oils were tested for their antifungal activity using the broth microdilution method according to Gong and Guo (Gong and Guo, 2009). The activity was tested against three pathogenic fungi: *Candida albicans* (MF942350), *Candida parapsilosis* (MF942354) and *Aspergillus parasiticus* (CBS 100926). One hundred microliters of the sample solutions was smeared in an SDA plate with approximately 3 × 10^6^ colony-forming units (CFU) mL^−1^, followed by incubation at 37 °C for 1 day. The zone of inhibition diameters (in mm) was measured, and the rates of growth inhibition were obtained according to the following formula taking into consideration ±SD as the means:

%Growth inhibition rate = (*d*_c_ − *d*_s_)/(*d*_c_ − *d*_0_) × 100

where *d*_c_ is the diameter of the untreated control fungus, *d*_s_ is the diameter of the sample-treated fungus and *d*_0_ is the diameter of the fungus cut.

The experiment was carried out in three independent replicates, and the result is the average with standard deviation. Itraconazole was used as the positive control, and DMSO was used as the negative control.

### 2.7 Statistical analysis

Data analysis was expressed as the mean ± standard deviation (SD) of three replicates. The data were subjected to one-way analysis of variance (ANOVA). Based on Microsoft Excel 2010 statistical package analyses, the significant differences were considered statistically significant values *p* < 0.05.

## 3 Results

**Figure 1:**
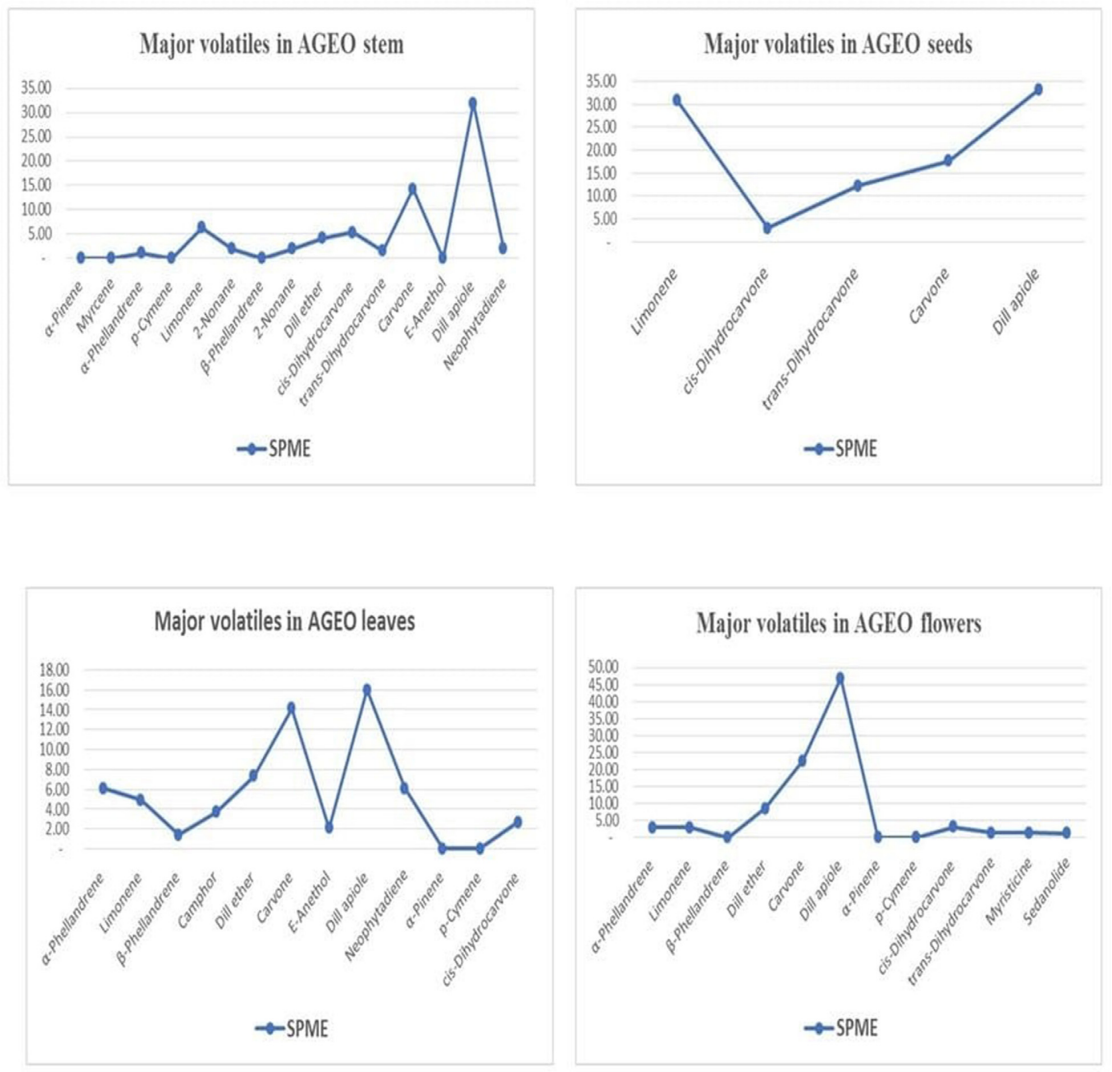
Peak areas of major volatiles in seeds, flowers, leaves and stems of *A. graveolens*.

Extracted volatile compounds from different parts of *A. graveolens* are tabulated in Table 1. Figs. S1-4 (a&b), which show the GC chromatograms of *A. graveolens* Essential oils of seeds, flowers, leaves and stems. The typical total ion chromatograms (TICs) of the seeds, flowers, leaves, and stem essential oils obtained by HS-SPME are shown in Figure 1 and indicate the differences in the volatile composition between different samples. Compounds are listed in order of their elution on the column HP-5 MS. Retention indices (RIs) calculated by GC capillary columns versus those obtained from the literature are also listed in the table.

**Table 1:** Composition of the flowers, seeds, leaves and stems of *A. graveolens* essential oils extracted using HS-SPME.

No.	Compounds	Rt (min.)	Flowers %	Seeds %	Leaves %	Stems %
1	*α*-Thujene	14.66	tr	tr	0.2	0.1
2	5-Methyl-3-heptanone	15.03	-	tr	-	0.3
3	*α*-Pinene	15.11	0.2	tr	0.4	0.2
4	Camphene	15.89	0.1	tr	0.1	tr
5	Benzaldehyde	16.16	tr	-	0.1	-
6	Sabinene	16.96	tr	tr	0.1	tr
7	*β*-Pinene	17.26	tr	tr	0.1	tr
8	Myrcene	17.54	0.1	0.1	0.3	0.2
9	Dehydro-1,8-cineole	17.75	-	-	-	-
10	*α*-Phellandrene	18.48	2.9	-	6.1	1.1
11	Verbenene	18.5	-	0.4	-	-
12	*α*-Terpinene	19.07	0.1	-	0.2	0.1
13	3-Carene	19.12	-	tr	-	-
14	*p*-Cymene	19.41	0.6	0.1	1.2	0.6
15	Limonene	19.70	3.0	30.8	4.9	6.3
16	*β*-Phellandrene	19.78	0.7	-	1.4	-
17	*β*-Ocimene	20.38	-	-	Tr	-
18	Artemisia ketone	20.96	0.1	tr	0.2	0.4
19	*γ*-Terpinene	21.17	tr	tr	0.3	0.1
20	Octanol	21.3	tr	-	Tr	-
21	*cis*-Sabinene hydrate	21.57	0.1	-	0.5	tr
22	2-Nonanone	22.41	0.3	tr	0.8	1.9
23	*p*-Cymenene	22.66	-	0.2	-	0.2
24	Terpinolene	22.65	0.1	-	0.2	-
25	Linalool	22.92	0.1	-	1.0	0.3
26	Undecane	22.96	-	tr	-	-
27	Nonanal	23.07	tr	-	Tr	0.2
28	Phenyl ethyl alcohol	23.77	-	-	0.1	-
29	*cis*-*p*-Menth-2-en-1-ol	24.33	-	-	-	-
30	*cis*-Limonene oxide	24.9	tr	0.1	-	-
31	*trans*-Limonene oxide	25.08	tr	tr	-	-
32	*trans*-*p*-Menth-2-en-1-ol	25.17	-	-	-	-
33	Camphor	25.61	0.5	tr	3.7	0.6
34	Menthone	25.93	-	-	-	-
35	Isomenthone	26.44	-	-	-	-
36	Borneol	26.68	tr	-	0.1	0.1
37	Terpinen-4-ol	27.22	tr	-	0.1	-
38	*p*-cymen-8-ol	27.31	-	-	-	-
39	Dill ether	27.58	8.5	0.1	7.3	4.1
40	*α*-Terpineol	27.77	-	tr	Tr	tr
41	Estragol	27.97	-	-	-	-
42	*cis*-Dihydrocarvone	28.02	3.1	3.0	2.7	5.3
43	*trans*-Dihydrocarvone	28.40	1.4	12.2	0.7	1.5
44	Dihydrocarveol isomer I	28.82	-	-	-	-
45	*iso*-Dihydrocarveol	28.84	0.2	-	-	0.3
46	Dihydrocarveol	28.87	tr	0.1	0.1	0.1
47	Cumin aldehyde	28.95	-	-	0.2	0.1
48	Carveol	29.03	-	0.1	-	-
49	Dihydrocarveol isomer II	29.49	-	-	-	-
50	*neoiso*-Dihydrocarveol	29.52	0.4	0.2	0.1	0.5
51	Fenchyl acetate	29.87	-	-	0.2	-
52	Pulegone	30.07	-	-	-	-
53	Carvone	30.35	22.6	17.7	14.1	14.3
54	Carvotanacetone	30.48	0.1	-	0.2	-
55	Piperitone	30.78	tr	-	Tr	-
56	Isopiperitenone	31.55	-	0.1	-	-
57	Carvone oxide	31.75	tr	tr	-	-
58	*E*-Anethol	32.12	0.2	tr	2.1	-
59	2-Undecanone	32.18	-	-	-	0.5
60	Bornyl acetate	32.26	0.1	-	1.0	0.1
61	Carvacrol	32.63	0.1	tr	0.6	0.7
62	Sabinyl acetate	34.13	tr	-	-	-
63	Terpinyl acetate	35.02	-	tr	-	0.1
64	*Z*-Ethyl cinnamate	36.07	0.1	-	0.4	tr
65	*E*-Methyl cinnamate	36.51	0.1	-	0.2	-
66	*α*-Ylangene	36.71	-	-	-	-
67	Methyl eugenol	37.12	-	-	Tr	-
68	*β*-Elemene	37.27	tr	-	0.1	-
69	E-β-Caryophyllene	38.78	0.1	-	0.9	0.3
70	*E-β*-Farnesene	39.56	tr	-	0.3	0.1
71	Dimethyltetrahydrobenzofuranone	39.85	0.1	-	1.0	-
72	*E*-Ethyl cinnamate	39.99	0.2	-	1.2	0.1
73	α-Humulene	40.23	-	-	0.1	-
74	Germacrene D	41.32	0.1	-	0.3	-
75	*β-*Selinene	41.62	tr	-	0.3	tr
76	Myristicin	42.40	1.3	0.2	0.9	0.9
77	*γ*-Cadinene	42.56	tr	-	0.1	tr
78	Dihydroactinidiolide	42.68	-	-	0.1	-
79	*δ*-Cadinene	42.8	-	-	0.2	-
80	Elemicin	43.37	tr	0.2	0.3	0.1
81	*E*-Nerolidol	43.97	0.1	-	0.7	0.4
82	*cis*-Davanone	44.98	-	-	0.9	-
83	Spathulenol	45.25	-	-	0.1	-
84	Caryophyllene oxide	45.57	-	-	0.1	-
85	Dill apiole	46.77	46.9	33.3	16.0	31.9
86	Butylphthalide	47.82	0.1	-	0.1	-
87	Apiol	48.64	0.1	tr	-	-
88	Sedanenolide	50.42	0.1	-	0.1	-
89	Sedanolide	51.41	1.2	tr	1.0	0.7
90	*neo*-phytadiene	54.09	0.5	tr	6.1	2.1
91	Phytone	54.25	tr	-	0.1	0.2
92	Methyl palmitate	56.87	tr	tr	0.1	0.1
93	Ethyl palmitate	59.20	-	tr	-	0.5
Total (%)			96.6	98.9	83.1	77.7
Monoterpene hydrocarbons			12.3	46.8	18.9	15.7
Oxygenated Monoterpenes			24.2	18.3	22.0	17.1
Sesquiterpene hydrocarbone			0.7	0	8.4	2.5
Oxygenated Sesquiterpene			0.1	0	1.8	0.4
Fatty acid			0	0	0.1	0.6
Miscellaneous			59.3	33.8	31.9	41.4

Rt = Retention time (min.), RI = Retention indices, tr = trace (<0.05%).

∗The isolated compounds are listed following their order of elution from the HP-5 MS column.

The HS-SPME analysis of *A. graveolens* essential oil of seeds led to the identification of forty volatile compounds (Table 1 & Fig. S1), including monoterpenes (65.1%) and miscellaneous classes (33.8%). Five major and five minor volatile components accounted for approximately 98.2% of the total identified components. The most abundant compounds were dill apiole (33.3%), limonene (30.8%), carvone (17.7%), *trans*-dihydrocarvone (12.2%) and *cis-*dihydrocarvone (3.0%). The minor compounds were verbenene (0.4%), *p*-cymenene (0.2%), dihydrocarveol (0.2%), myristicine (0.2%) and elemicin (0.2%).

Moreover, the oil extract of the flowers was rich in oxygenated monoterpenes and monoterpene hydrocarbons (24.2% and 12.3%, respectively). Dill apiole (46.9%), carvone (22.6%), dill ether (8.5%), *cis*-dihydrocarvone (3.1%), limonene (3.0%), *α*-phellandrene (2.9%), *trans*-dihydrocarvone (1.4%), myristicine (1.3%), and sedanolide (1.2%) were reported as the main compounds among the sixty constituents identified in the flower essential oil, representing 96.6% of the total components detected. The minor compounds were *p*-cymenene (0.6%), camphor (0.5%), *neo*-phytadiene (0.5%), *neo-iso*-dihydrocarveol (0.4%), 2-nonanone (0.3%), *α*-pinene (0.2%), *iso*-dihydrocarveol (0.2%), (*E*)-anethol (0.2%) and (*E*)-ethyl cinnamate (0.2%), as shown in Table 1 & Fig. S2.

As indicated in Table 1 & Fig. S3, sixty-six components representing 83.1% of the total content were identified in *A. graveolens* essential oil leaves included 40.9% monoterpenes and 10.2% sesquiterpenes. Sixteen of them were found over a 1.0% peak area, while the rest of the constituents were in concentrations less than 1.0% peak area (Figure 1). The major constituents of the leaves of *A. graveolens* essential oils were as follows: dill apiole (16.0%), carvone (14.1%), dill ether (7.3%), *α*-phellandrene (6.1%), neophytadiene (6.1%), limonene (4.9%), camphor (3.7%), *cis*-dihydrocarvone (2.7%), *E*-anethol (2.1%), *β*-phellandrene (1.4%), *p*-cymene (1.2%), *E*-ethyl cinnamate (1.2%), dimethyltetrahydrobenzofuranone (1.0%), linalool (1.0%) and bornyl acetate (1.0%). The minor compounds that were found in the range of 0.2–0.9% peak area and represented approximately 10.4% were *E*-*β*-caryophyllene (0.9%), myristicin (0.9%), *cis*-davanone (0.9%), 2-nonanone (0.8%), *trans*-dihydrocarvone (0.7%), *E*-nerolidol (0.7%), carvacrol (0.6%), *cis*-sabinene hydrate (0.5%), *α*-pinene (0.4%), *Z*-ethyl cinnamate (0.4%), myrcene (0.3%), *γ*-terpinene (0.3%), *E*-*β*-farnesene (0.3%), germacrene D (0.3%), *β*-selinene (0.3%), elemicin (0.3%), *α*-thujene (0.2%), *α*-terpinene (0.2%), artemisia ketone (0.2%), terpinolene (0.2%), cumin aldehyde (0.2%), fenchyl acetate (0.2%), carvotanacetone (0.2%), *E*-methyl cinnamate (0.2%).

**Figure 2:**
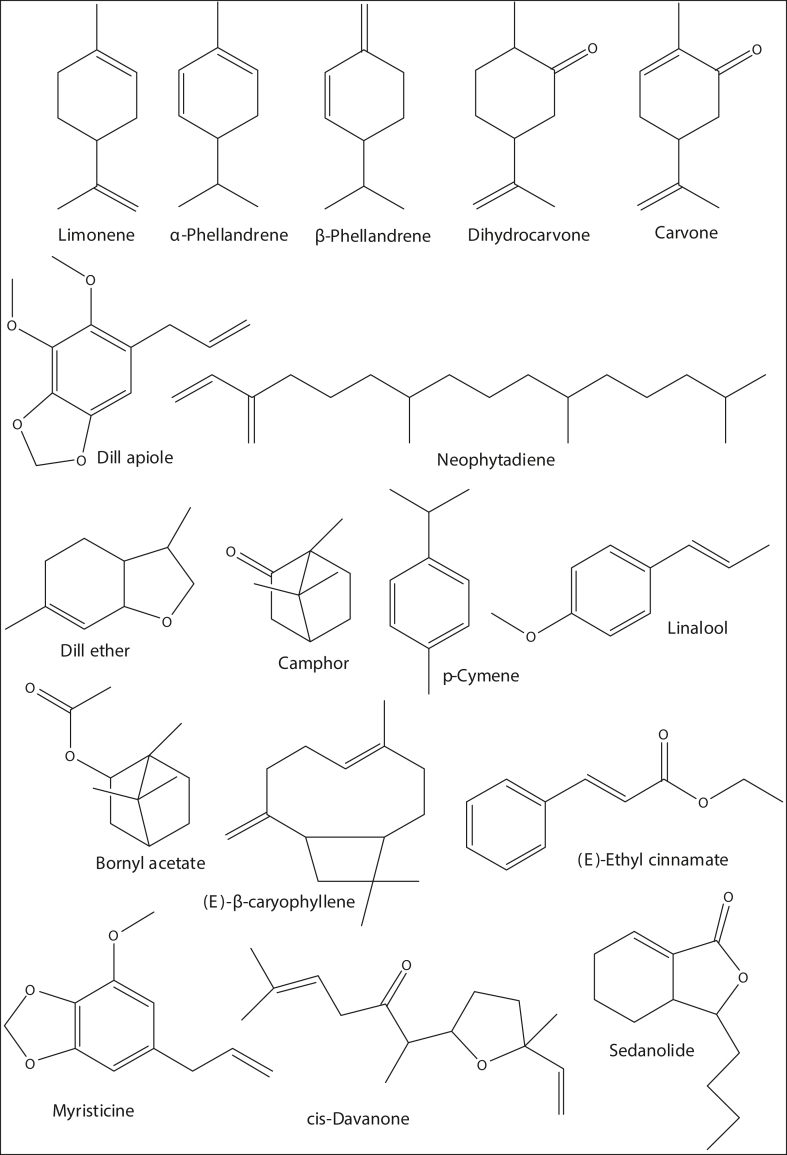
Structures of major constituents in essential oils of *A. graveolens* seeds, flowers, leaves and stems.

Furthermore, forty-eight components were extracted from the stems of *A. graveolens* essential oils (Table 1 & Figs. S4). *α*-Phellandrene, limonene, dill ether and dill apiole were the major compounds of stem oils; their percentages were 1.1%, 6.3%, 4.1% and 31.9%, respectively (Figure 1). However, myristicine (0.9%), carvacrol (0.7%), sedanolide (0.7%), *p*-cymene (0.6%), camphor (0.6%), *neo-iso*-dihydrocarveol (0.5%), 2-undecanone (0.5%), ethyl palmitate (0.5%), artemisia ketone (0.4%), *E*-nerolidol (0.4%), 5-methyl-3-heptanone (0.3%), linalool (0.3%), *iso*-dihydrocarveol (0.3%) and *E*-β-caryophyllene (0.3%) were the minor constituents found in the range of 0.3–0.9% peak area. Structures of major constituents present in the essential oils of *A. graveolens* seeds, flowers, leaves and stems are illustrated Figure 2.

The essential oils of *A. graveolens* samples (seeds, flowers, leaves and stems) were tested for antimicrobial activity against gram-positive, such as *S. aureus* (CP011526.1), *B. licheniformis* (KX785171.1), and *L. innocua* (DSM, 20649), and gram-negative, such as *E. xiangfangensis* (CP017183.1), *E. fergusonii* (CU928158.2) and *P. aeruginosa* (NR-117678.1). In addition to the three pathogenic fungi *C. albicans* (MF942350), *C. parapsilosis* (MF942354) and *A. parasiticus* (CBS 100926), as shown in Table 2. The oil from seeds at 20 μL exhibited the highest antimicrobial activity. The best activity was observed against the *S. aureus* strain, with a diameter of inhibition equal to 25.0 ± 0.00, 17.0 ± 0.10, and 15.0 ± 1.10 mm for the essential oil isolated from seeds, flowers and leaves, respectively. This strain had a lower sensitivity to the essential oil isolated from stems (11.0 ± 0.6 mm) compared to the antibiotic tetracycline. The halos of inhibition for *B. licheniformis* (22.0 ± 1.60 and 18.0 ± 1.90 mm) by the essential oils from seeds and flowers, respectively, were greater than those of the remaining samples. In the antifungal assay, the inhibitory actions of all the tested samples against *A. parasiticus* were significantly higher than that of the control antifungal drug itraconazole (Table 2).

**Table 2:** Antimicrobial of the *A. graveolens* essential oils of seeds, flowers, leaves and stems.

Microbial strains	Dose (μL)	Essential oil				Control		
		Seeds	Flowers	Leaves	Stems	Gentamicin	Tetracycline	Itraconazole
*S. aureus* CP011526	10	17.0 ± 0.80	15.0 ± 0.00	12.0 ± 1.40	8.0 ± 0.60	6.0 ± 0.00	15.3 ± 0.60	na
	20	25.0 ± 0.00	17.0 ± 0.10	15.0 ± 1.10	11.0 ± 0.60			
*B. licheniformis KX*785171	10	15.0 ± 1.30	12.0 ± 0.80	11.0 ± 1.40	8.0 ± 0.60	12.0 ± 1.00	18.2 ± 1.80	na
	20	22.0 ± 1.60	18.0 ± 1.90	12.0 ± 1.50	10.0 ± 1.30			
*L. innocua* DSM 20649	10	11.0 ± 1.30	9.0 ± 1.20	7.0 ± 0.90	7.0 ± 0.10	26.3 ± 0.40	21.2 ± 1.80	na
	20	25.0 ± 0.96	23.0 ± 1.70	19.0 ± 2.30	15.0 ± 1.30			
*E. xiangfangensis* CP017183	10	11.0 ± 0.31	9.0 ± 0.06	8.0 ± 11.0	5.0 ± 0.29	8.0 ± 0.00	15.0 ± 0.60	na
	20	13.0 ± 0.82	12.0 ± 0.12	10.0 ± 0.08	7.0 ± 0.23			
*E. fergusonii* CU928158	10	6.0 ± 1.50	6.0 ± 0.00	4.0 ± 0.60	2.0 ± 1.70	7.0 ± 8.00	10.0 ± 1.0	na
	20	8.0 ± 1.20	7.0 ± 1.00	5.0 ± 0.90	3.0 ± 1.00			
*P .aeruginosa* NR117678	10	4.0 ± 1.50	2.0 ± 1.60	3.0 ± 1.70	3.0 ± 0.40	11.0 ± 0.60	15.0 ± 1.20	na
	20	8.0 ± 1.80	5.0 ± 2.30	4.0 ± 1.20	3.0 ± 1.50			
*C. albicans* MF942350	10	16.0 ± 0.59	13.0 ± 0.78	11.0 ± 1.38	7.0 ± 1.52	na	na	15.0 ± 1.70
	20	18.0 ± 0.80	16.0 ± 0.90	13.0 ± 0.88	10.0 ± 1.08			
*C. parapsilosis* MF942354	10	18.0 ± 2.30	12.0 ± 1.10	9.0 ± 1.00	9.0 ± 0.84	na	na	17.0 ± 2.00
	20	20.0 ± 1.90	15.0 ± 0.72	11.0 ± 1.80	10.0 ± 0.78			
*A. parasiticus* CBS 100926	10	25.0 ± 0.14	21.0 ± 0.80	19.0 ± 0.70	11.0 ± 0.14	na	na	19.0 ± 1.60
	20	42.0 ± 0.30	33.0 ± 0.54	25.0 ± 0.23	22.0 ± 1.50			

Data represent the diameter inhibition (in mm). Results are the mean of three repetitions ±standard deviation (SD) of the inhibition zone. na = not active.

## 4 Discussion

GC–MS analyses were conducted for the essential oils that were obtained from different parts of *A. graveolens* growing in Saudi Arabia. Myrcene, *α*-phellandrene, *p*-cymene, limonene, dill ether, carvone and dill apiole were found to be common components of *A. graveolens* plant seeds, flowers, leaves and stems essential oils. Dill ether is the most abundant constituent of *A. graveolens* essential oils, and it is found in sufficient quantities in all parts; 46.9, 33.3, 16.0, and 31.9% in flowers, seeds, leaves, and stems, respectively. The second main volatile component of *A. graveolens* essential oils were *α*-phellandrene, which was found in adequate quantities in flowers (2.9%), seeds (traces), leaves (6.1%) and stems (1.1%).

It is well established that essential oils are generally effective against many microorganisms, including bacteria and fungi. As typical lipophiles, they disrupt the structure of the cytoplasmic membrane and permeabilize it. In bacteria, membrane permeabilization is associated with the loss of ions and reduction of membrane potential (Bakkali et al., 2008; Sikkema et al., 1994). In this context, the present work was carried out to compare and investigate the activity of *A. graveolens* essential oils isolated from different parts against various strains of bacteria and fungi, the positive results will confirm the folkloric use of *A. graveolens* for curing many infectious diseases.

Our results show that *A. graveolens* oil samples (seeds, flowers, leaves and stems) exhibited good antimicrobial activities against the tested microorganisms (Table 2). Data analysis showed that the essential oil isolated from *A. graveolens* seeds exhibited the highest antimicrobial activity compared to other essential oils isolated from other parts of the same plant, followed by flowers, leaves and stems. Previous findings showed that essential oils rich with carvone and limonene usually exhibit strong antimicrobial activity (Delaquis et al., 2002; Singh et al., 2002). Taking this into account, one can expect good antimicrobial potential of *A. graveolens* essential oil in which carvone and its precursor limonene are the major constituents. This was proven by the antimicrobial assay of these samples, in which they showed superior activities when compared to control antimicrobials. The highest activity was observed against the *S. aureus* strain, with a diameter of inhibition equal to 25.0 ± 0.00 mm, followed by *B. licheniformis,* with a diameter of inhibition of 22.0 ± 1.60 mm, compared to gentamicin and tetracycline. Interestingly, all tested *A. graveolens* essential oil samples showed stronger antifungal activity against *A. parasiticus* than the control antifungal itraconazole (Table 2). These results are consistent with data previously reported that essential oils extracted from dill plants exhibit antifungal activity (Kaivan et al., 2016; Yuxin et al., 2013; Jun et al., 2012). To our knowledge, this is the first report with comparable testing of different parts of Saudi *A. graveolens* essential oils (seeds, flowers, leaves and stems) against different pathogenic microorganisms.

## 5 Conclusions

In this study, the chemical profile and antimicrobial activities of essential oils from the different parts of Saudi *A. graveolens* were analyzed. HS-SPME was applied as an extraction method for volatiles. Dill ether is the most abundant constituent of *A. graveolens* essential oils, and it was found in sufficient quantities in all studied parts of the plant. A comparison of different parts revealed that *A. graveolens* seed oils exhibited the most potent antimicrobial activity when compared to the other parts of the plant. This could be attributed to the presence of the highest content of carvone and its precursor limonene in seed essential oil. To our knowledge, this study is the first to evaluate and compare different parts of *A. graveolens* plants growing in Saudi Arabia in terms of their chemical composition and antimicrobial activities.

## Declarations

### Author contribution statement

Hanan Y. Aati; Shagufta Perveen: Conceived and designed the experiments; Performed the experiments; Analyzed the data; Wrote the paper.

Sultan Aati; Abdulrahman Y. Aati: Contributed reagents, materials, analysis tools or data; Wrote the paper.

Raha Orfali: Performed the experiments.

Jawaher H. Alqahtani; Areej M. Al-Taweel: Analyzed and interpreted the data; Editing the paper.

Juergen Wanner: Performed the experiments; Analyzed the data.

### Funding statement

This work was supported by Researchers Supporting Project (RSP2022R504), King Saud University, Riyadh, Saudi Arabia.

### Data availability statement

Data will be made available on request.

### Declaration of interests statement

The authors declare no conflict of interest.

### Additional information

No additional information is available for this paper.
