# Supplementary material for: Headspace solid-phase microextraction method for extracting volatile constituents from the different parts of Saudi Anethum graveolens L. and their antimicrobial activity
Source: Heliyon. 2022 Mar 5;8(3):e09051. doi: 10.1016/j.heliyon.2022.e09051 (PMC8920927; doi:10.1016/j.heliyon.2022.e09051)
Supplement: Supplementary_material [file mmc1.doc]

**Supplementary materials**

**Headspace solid-phase microextraction method for extracting volatile constituents from the different parts of Saudi *Anethum graveolens* L. and their antimicrobial activity**

Hanan Y. Aati^a*^, Shagufta Perveen^b*^, Sultan Aati^c^, Raha Orfali^a^, Jawaher H. Alqahtani^a^, Areej M. Al-Taweel^a^, Juergen Wanner^d^, Abdulrahman Y. Aati^e^

*^a^Department of Pharmacognosy, College of Pharmacy, King Saud University. P.O. Box 2457, Riyadh 11451, Saudi Arabia*

*^b^Department of Chemistry, School of Computer, Mathematical and Natural Sciences, Morgan State University, Baltimore, MD 21251, USA*

*^c^UWA Dental School, 17Monash Avenue, Nedland WA 6009, Australia*

*^d^Kurt Kitzing Co. Hinterm Alten Schloss 21, D-86757 Wallerstein, Germany*

*^e^Rokn Al-Madaein Pharmaceutical Warehouse Co., Riyadh, Kingdom of Saudi Arabia, Abdulrahman11aati@gmail.com*

^*^Correspondence: Hanan Y. Aati; [hati@ksu.edu.sa](mailto:hati@ksu.edu.sa); ORCID# 0000-0003-1598-2273

Shagufta Perveen; shagufta792000@yahoo.com

**
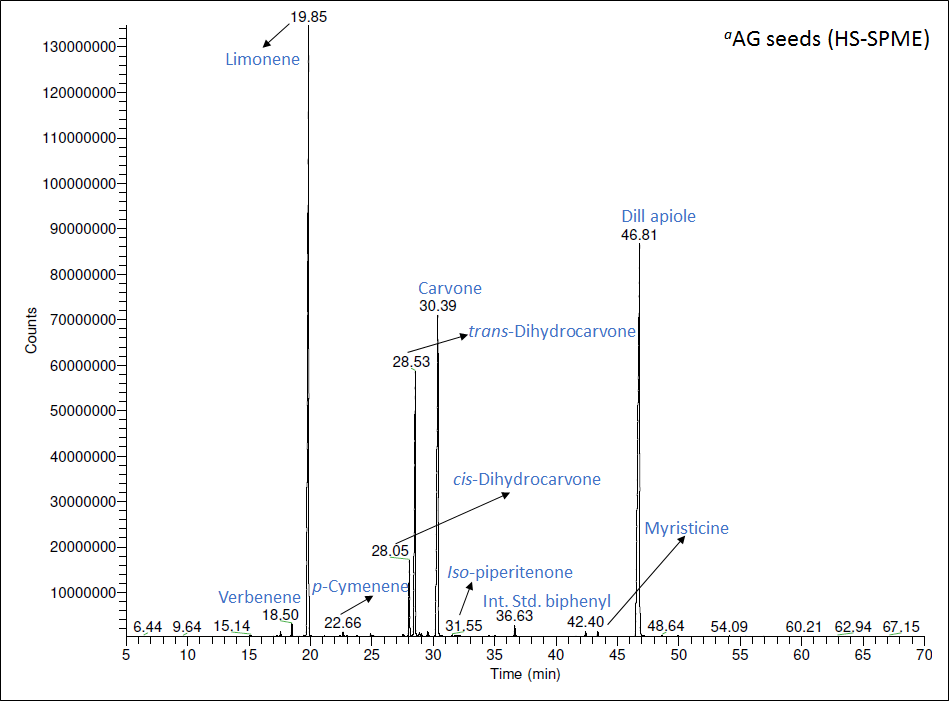
**

**Fig. S1** Typical GC-FID chromatographic profiles of *A.* [*graveolens*](https://www.sciencedirect.com/topics/food-science/dill) essential oil obtained from seeds (HS-SPME)

**
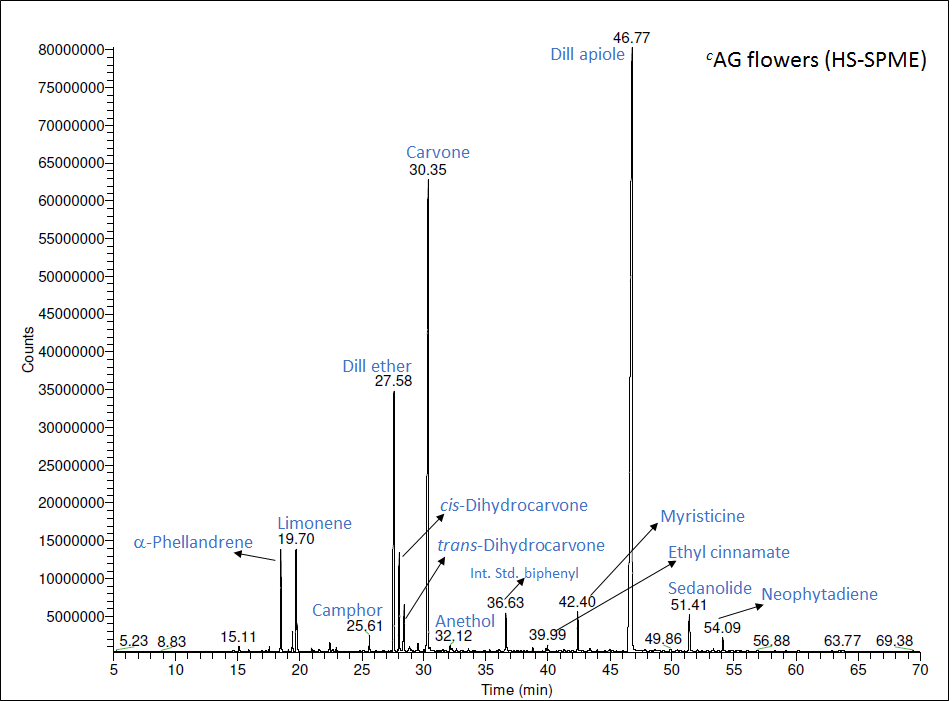
**

**Fig. S2** Typical GC-FID chromatographic profiles of *A.* [*graveolens*](https://www.sciencedirect.com/topics/food-science/dill) essential oil obtained from flowers (HS-SPME)

**
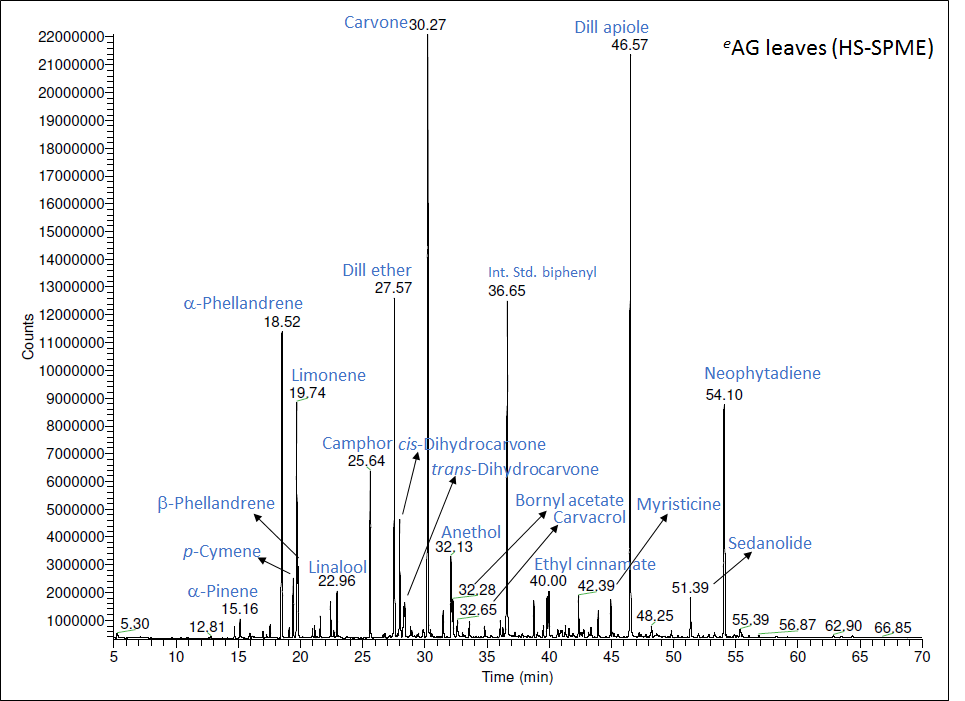
**

**Fig. S3** Typical GC-FID chromatographic profiles of *A.* [*graveolens*](https://www.sciencedirect.com/topics/food-science/dill) essential oil obtained from leaves (HS-SPME)

**
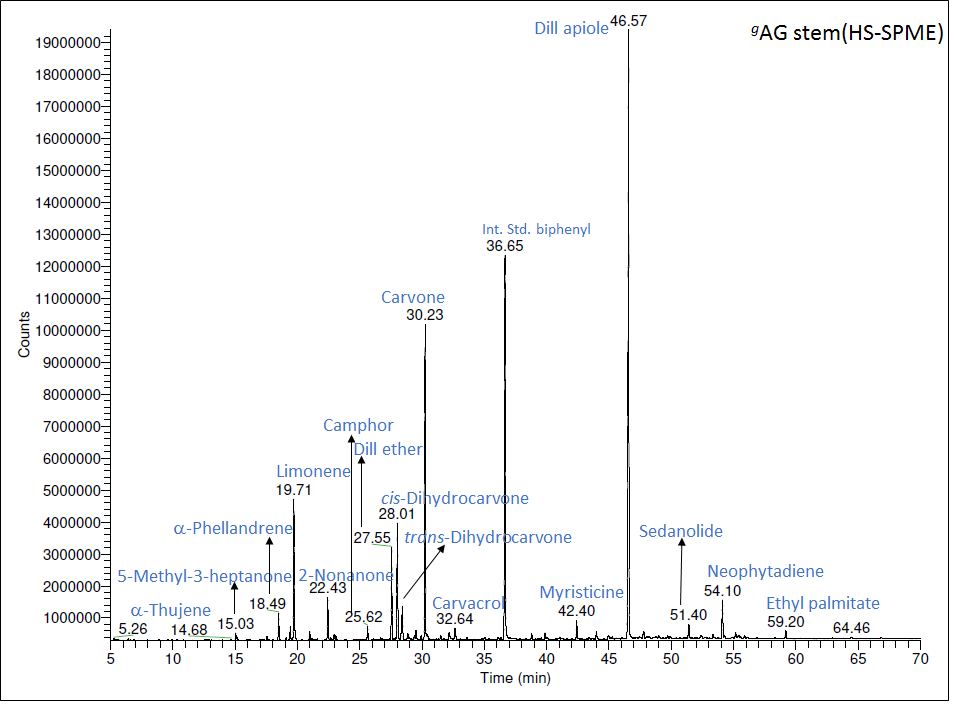
**

**Fig. S4** Typical GC-FID chromatographic profiles of *A.* [*graveolens*](https://www.sciencedirect.com/topics/food-science/dill) essential oil obtained from stem (HS-SPME)
